# Construction Notes

**Published:** 1894-03-31

**Authors:** 


### CONSTRUCTION NOTES.

### New Cottage Hospital at Clun.

This hospital, the gift of Mrs. Henry Darrell Brown, of White Rock House, Hastings, was formally opened recently. As stated in the rules, the establishment is intended for the accommodation of poor persons resident in the parishes of Clun, Clunbury, Newcashe, and Bethos Y-Crewyn. The interior contains two spacious wards, fitted up with hot and cold water, the different apartments being furnished under the immediate superintendence of the founder. The cost of the site was £500, and it has been stated that the amount of the endowments would probably be between £10,000 and £12,000. The arehitest was Mr. F. R. Kempson, and the contractors Messrs. W. C. Cooke and J. Anthony, of Clun, whose tender was accepted for £1,232.

### City of Dublin Hospital Extensions.

The improvements in Baggot Street Ho pital, amounting almost to the erection of a structure double the size of the old building, are fast approaching completion, and in a few months the public may expect to see fully realised the great and necessary work, for the accomplishment of which the Earl of Pembroke and the promoters of the Kosmic bazaar deserve so much credit. Briefly, what has been done may be summarised by saying that the front of the hospital has been brought out about 20 feet from its former site, the height of the entire building has been increased by an additional storey, the height of the front portion being increased by two new storeys; and a new wing, 25 feet wide and 80 feet in length, has also been added on to the northern side of the hospital. The different floors on each storey have been placed on the same level, and a series of corridors have been constructed, running north and south, bisecting the building on each storey, and affording facilities for ventilation and for locomotion that until now were unknown. A commodious stone staircase has been constructed in the front portion of the building, but in addition to this several elevators have also been provided. All the sanitary arrangements have been renewed and made as perfect as modern science could make them. Improved accommodation for the nursing staff, and for the Matron and Lady Superintendent, as well as for the house surgeon and the resident pupils, has been provided. A hot-air chamber has been constructed for drying linen; and all linen to be washed will be shot down a tube to the sorting room below, whence it will be sent to the laundry. The plumbing and sanitary work has been looked after by Mr. William Baird, of Abbey Street, and the heating apparatus has been supplied by Messrs. Bacon and Co., of London. The architectural work in connection with the new buildings has been done by Mr. Albert E. Murray, of Dawson Street, Dublin.

